# Prophylaxis of post-ERC infectious complications in patients with biliary obstruction by adding antimicrobial agents into ERC contrast media- a single center retrospective study

**DOI:** 10.1186/s12876-017-0570-4

**Published:** 2017-01-13

**Authors:** Hella Wobser, Agnetha Gunesch, Frank Klebl

**Affiliations:** 1 Department of Internal Medicine and Gastroenterology, University Hospital of Regensburg, Regensburg, 93042 Germany; 2 Present address: Praxiszentrum Alte Mälzerei, Regensburg, Germany

**Keywords:** Endoscopic retrograde cholangiography (ERC), Intraductal antimicrobial prophylaxis, Infectious complications, Biliary obstruction, Secondary sclerosing cholangitis

## Abstract

**Background:**

Patients with biliary obstruction are at high risk to develop septic complications after endoscopic retrograde cholangiography (ERC). We evaluated the benefits of local application of antimicrobial agents into ERC contrast media in preventing post-ERC infectious complications in a high-risk study population.

**Methods:**

Patients undergoing ERC at our tertiary referral center were retrospectively included. Addition of vancomycin, gentamicin and fluconazol into ERC contrast media was evaluated in a case-control design. Outcomes comprised infectious complications within 3 days after ERC.

**Results:**

In total, 84 ERC cases were analyzed. Primarily indications for ERC were sclerosing cholangitis (75%) and malignant stenosis (9.5%). Microbial testing of collected bile fluid in the treatment group was positive in 91.4%. Detected organisms were sensitive to the administered antimicrobials in 93%. The use of antimicrobials in contrast media was associated with a significant decrease in post-ERC infectious complications compared to non-use (14.3% vs. 33.3%; odds ratio [OR]: 0.33, 95% confidence interval [CI]: 0.114–0.978). After adjusting for the variables acute cholangitis prior to ERC and incomplete biliary drainage, the beneficial effect of intraductal antibiotic prophylaxis was even more evident (OR = 0.153; 95% CI: 0.039–0.598, *p* = 0.007). Patients profiting most obviously from intraductal antimicrobials were those with secondary sclerosing cholangitis.

**Conclusion:**

Local application of a combination of antibiotic and antimycotic agents to ERC contrast media efficiently reduced post-ERC infectious events in patients with biliary obstruction. This is the first study that evaluates ERC-related infectious complications in patients with secondary sclerosing cholangitis. Our first clinical results should now be prospectively evaluated in a larger patient cohort to improve the safety of ERC, especially in patients with secondary sclerosing cholangitis.

## Background

Infections such as cholangitis and sepsis are serious, albeit rare complications after endoscopic retrograde cholangioscopy (ERC). Post-ERC infections are reported to occur in less than 5% of all interventions [1, 2]. High hygienic standards for the intervention itself and thorough disinfection and storage of endoscope and endoscopic devices have essentially attributed to this low infectious rate [3]. Procedural improvements such as endoscopic decompression by biliary stents and immediate placement of percutaneous biliary drainage if endoscopic drainage is not possible, represent further strategies to reduce the incidence of ERC-related infectious complications [4, 5]. This is an important issue, as failure to restore an adequate drainage after injection of contrast media into obstructed bile tracts during ERC still represents the major risk factor for post-ERC infection [6, 7].

Obstruction of the bile duct system due to stones, strictures and tumors has been demonstrated to be associated with bacteriobilia [8]. Increasing intrabiliary pressure (>25 mmHg) results in biliovenous reflux and consecutively in bacteremia in case of already infected bile [9, 10]. Injection of contrast media during ERC raises the intraductal pressure, especially if a complete endoscopic drainage is not achieved thereafter. Therefore, patients with hilar tumors and sclerosing cholangitis for whom complete biliary drainage is often impossible, are at highest risk to develop post-ERC infections [11, 12].

Routine prophylactic use of systemic antibiotics was shown to reduce ERC-related bacteremia [13]. However, beneficial effects on preventing post-ERC cholangitis in unselected patients could not be demonstrated [14–16]. A recent retrospective study analyzed the benefit of systemic antibiotic prophylaxis in 11.484 patients undergoing ERC over an 11-year period [17]. At baseline all patients with biliary obstruction, immunosuppression and the need of therapeutic intervention (95% of all procedures) received routinely systemic prophylactic antibiosis. Over time, the use of prophylactic antibiotics was sequentially reduced. In the final phase, systemic antibiotic prophylaxis was restricted to patients for whom endoscopic drainage was predicted to be incomplete and to patients with immunosuppressive therapy (26% of all procedures). Despite the limited use of systemic antibiotic prophylaxis, no significant difference in infectious complications after ERC was observed. These data are in line with the current recommendations of antibiotic prophylaxis in gastrointestinal endoscopy [18, 19]. Systemic antibiotic prophylaxis should be considered before an ERC in those patients with known or suspected biliary obstruction for whom complete endoscopic drainage will presumably not be achieved. This concerns especially patients with hilar strictures and primary sclerosing cholangitis (PSC).

Of note, biliary excretion of systemically administered antibiotic agents was shown to be low in case of biliary obstruction or hepatic dysfunction [20]. Thus, antibiotic bile concentrations may be far below the minimal inhibitory concentration (MIC). Theoretically, local application of antibiotics into the ERC contrast media should result in high antibacterial concentration within the bile. Thus, this regimen is supposed to be especially effective in preventing ERC-related cholangiosepsis. Indeed, in vitro studies have demonstrated that addition of gentamicin to the ERC contrast media eliminated bacteriobilia [21]. In a high-risk study population, the combination of intravenous and intraductal antibiotic administration was shown to efficiently prevent post-ERC infectious complications [22]. Most recently, adding gentamicin to contrast media had no significant effect on the incidence of post-ERC cholangitis, however adequate drainage of biliary obstruction by stenting was obtained in all these patients [23].

Taking these rather heterogeneous and inconsistent data into account, we aimed to evaluate whether local application of antimicrobial agents into contrast media will be beneficial to reduce post-ERC infectious complications in a study population mainly predicted to incomplete endoscopic drainage.

## Methods

### Study population

#### Data acquisition

This retrospective single-center study covers an 8-year-period from January 2003 to December 2011. During this time, 1353 patients with biliary obstruction underwent ERC. Of these, 101 patients received antimicrobial agents into the ERC contrast media. 59 patients with incomplete follow up or with ERC within the preceding 70 days were excluded from this study. 13 patients underwent ERC with similar indication twice within 5 years with and without intraductal antibiotics, respectively. These were included as case- and control-ERCs into our study. 29 patients with antibiotic application into the contrast media during ERC were matched to 29 control patients without antibiotic administration in respect to indication of ERC, age and sex. In summary, our study encompasses 84 ERC cases with 42 cases receiving antibiotics into the ERC contrast media and 42 control cases without antibiotics.

#### Demographic data

Mean age of the predominantly male (71%) study population was 52 +/- 16.2 years. All patients presented with biliary obstruction. Malignant strictures (cholangiocellular carcinoma [*n* = 5], pancreatic cancer [*n* = 2], metastasis [*n* = 1]) and sclerosing cholangitis (primary sclerosing cholangitis [*n* = 20] and secondary sclerosing cholangitis [*n* = 44]) were the most prevalent causes of biliary obstruction. Other etiologies of obstructive bile tract system included choledocholithasis (*n* = 4), benign stenosis after liver transplantation (*n* = 2), acute cholangitis due to stent obstruction (*n* = 3) or benign stricture (*n* = 1) and chronic cholangitis (*n* = 2). Thus, the study population was mainly composed of high-risk patients regarding infectious post-ERC adverse events.

### Definition of ERC-related infectious complications

In case of absent non-/biliary infection by the time of ERC (a) a rise in body temperature > 38 °C within 24 h after ERC (in case of body temperature < 38 °C before ERC) or (b) increase of white blood cell count and/or CRP over upper normal limits in combination with elevation of transaminases (Δ10 U/l) and bilirubin (Δ1.5 mg/dl) within 3 days after ERC were defined as infectious complication.

When non-/biliary infection was present by the time of ERC, (c) a rise in body temperature > 38 °C within 24 h after ERC (incase of body temperature < 38 °C before ERC) or (d) increase of white blood cell count of Δ2000/μl within 3 days after ERC or (e) increase of CRP Δ50 mg/l within 3 days of ERC characterized infectious complication.

### Definition of successful ERC

ERC was categorized as successful when (a) biliary drainage was restored and laboratory tests for alkaline phosphatase, γ-glutamyltransferase and bilirubin as well as transaminases decreased after ERC, (b) in case of sclerosing cholangitis: laboratory tests for alkaline phosphatase, γ-glutamyltransferase and bilirubin as well as transaminases decreased after ERC, even if complete biliary decompression failed, and (c) in case of stent removal/replacement: laboratory tests for alkaline phosphatase, γ-glutamyltransferase and bilirubin as well as transaminases remained at least stable.

### Statistical analysis

All statistical analyses were performed with SPSS Version 22 (SPSS Inc., Chicago, IL, USA). Descriptive data of patients are presented as mean values with the interquartile range for continuous variables or percentage for categorial variables. Pearsons’s chi-squared test was used to compare categorial data. Factors influencing the risk of post-ERC infectious complications were analyzed using binary logistic regression models. Due to the low patient numbers, it was predefined that only the two presumably most important risk factors for infectious complications, namely presence of acute cholangitis at ERC, and incomplete biliary drainage, would be included in the multivariate logistic regression to calculate the effect of intraductal administration of antimicrobial agents on post-ERC infectious complications. Values of *p* <0.05 were considered to be statistically significant.

## Results

### Patient demographics and clinical features

Eighty-four cases of biliary obstruction undergoing ERC in our tertiary referral center were analyzed in this retrospective study to evaluate the benefit of antimicrobial agents added to the contrast media on the rate of post-ERC infectious complications. Therefore, 42 cases receiving antibiotics into ERC contrast media were matched to 42 controls without antibiotics for the parameters age, sex and etiology of biliary obstruction. Patient characteristics are shown in Table 1 for both groups.

**Table 1:** Clinical characteristics of the study population

Patient characteristics	Intraductal antibiotic prophylaxis	No antibiotic prophylaxis	*p*
Mean age	51.8 ± 16.4	52.4 ± 16.2	0.26
Male (*n*; %)	30 (71)	30 (71)	
Etiology of biliary obstruction (*n*; %)			
• Sclerosing cholangitis	32 (76)	32 (76)	
• Malignant stricture	4 (10)	4 (10)	
• Choledocholithiasis	2 (5)	2 (5)	
• Benign stricture	1 (2)	1 (2)	
• Cholangitis	3 (7)	3 (7)	
Immunosuppressive medication (*n*; %)	11 (26)	14 (33)	0.49
Hospitalization (d)	13.9 ± 21.1	13.3 ± 16.6	0.47
Cholangitis at ERC (*n*; %)	27 (64)	23 (55)	0.37
Non-biliary infection (*n*; %)	16 (38)	13 (31)	0.49

Patients were matched in respect to age, sex and indication for ERC

In the treated group (*n* = 42) the following antimicrobial agents were administered to 50 ml contrast media (Optiray 300 g/ml): gentamicin 80 mg (2 ml), vancomycin 500 mg (5 ml) and fluconazole 40 mg (20 ml). Most patients in the treated group received a combination of all antimicrobial agents (*n* = 29; 69% of treated cases). Solely gentamicin was given in 7 cases (16.7%), whereas a combination of both antibiotics was administered in 6 cases (14.3%). In addition, 51.3% (43/84) of all patients received a systemic antibiotic treatment within 28 days prior to and at ERC. Of note, there was no statistical difference between the two study groups regarding frequency of systemic antibiotic treatment (24/42 patients in the treated group vs. 19/42 in the control group, *p* = 0.28). Most frequently, patients with secondary sclerosing cholangitis (SC) [29 out of 44 SC-patients, 65.9%], with primary sclerosing cholangitis (PSC) [6 out of 20 PSC-patients, 30%] and with choledocholithiasis [3 out of 4 patients, 75%] received systemic antibiotic treatment prior to and at ERC. The main indication for antibiotic treatment was acute cholangitis.

Details on ERC data are shown in Table 2. There was no statistical difference between the two groups regarding the endoscopic procedures.

**Table 2:** Details of selected endoscopic procedures

Procedure	Intraductal antibiotic prophylaxis	No antibiotic prophylaxis	*p*
Papillotomy	16	11	0.24
Stone removal	6	3	0.29
Necrosis removal	3	1	0.3
Dilatation	12	5	0.06
Lavage	4	3	0.69
Nasobiliary drain	5	3	0.46
Stenting			0.84
• Stent insertion	4	4	
• Stent exchange	3	2	
• Stent removal	2	3	

### Microbial cultures of bile samples

Thirty-five bile samples (83.3% of the treated cases) taken from patients receiving antimicrobial agents into the contrast media were analyzed on microbial colonization (Table 3). Only three bile cultures (8.6%) were tested negative for bacterial and fungal species. The most frequently isolated bacterial organisms in the collected bile samples were *Enterococcus* spp. found in 71.4% (25/35), *E. coli* in 25.7% (9/35), *Klebsiella* spp. in 11.4% (4/35), *Pseudomonas* spp. in 11.4% (4/35) and other gram-negative bacteria in 11.4% (4/35). *Candida* spp. were isolated in 25.7% (9/35) of the bile samples. Polymicrobial infection was detected in 54% (19/35) of bile samples. The results of the antibiogram were not available for 7 bile cultures. In 3 bile samples the isolated bacteria were resistant to the administered intraductal antibiotics. All *Candida species* were sensitive to fluconazole.

**Table 3:** Bile cultures of bile samples from patients receiving antimicrobial agents into ERC contrast media

Cultures positive for	*n* (%)
*Enterococcus* spp.	25 (71.4%)
*E. coli*	9 (25.7)
*Klebsiella* spp.	4 (11.4)
*Pseudomonas* spp.	4 (11.4)
*Candida* spp.	9 (25.7)
Other gram-negative	4 (11.4)
Polymicrobial	19 (54.0)

Note, that the sum of percentages may be greater than 100 because of polymicrobial infections

### Antimicrobial agents in ERC contrast media reduced ERC-related infectious complications

**Fig. 1:**
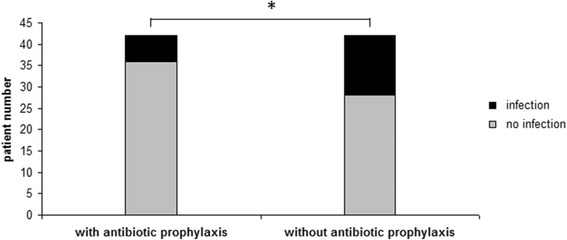
ERC-related infectious complications. Patients with antibiotic prophylaxis within the contrast media developed post-ERC infectious complications significantly less frequent than patients not receiving antimicrobial agents (14.3% versus 33.3%, *p* < 0.04)

ERC-related infectious complications were observed in 23.8% of patients (20/84). While 33.3% (14/42) of patients not subjected to antimicrobial agents into the contrast media developed a post-ERC infectious complication, only 14.3% (6/42) patients receiving antibiotics within the ERC contrast media presented with signs and symptoms of infection (OR = 0.33, 95% CI 0.114–0.978; *p* < 0.04; Fig. 1). Hence, the risk to develop an infectious complication after ERC was 2.33-fold higher when ERC was performed without administering antimicrobial agents to the contrast media.

Among the 20 patients with post-ERC infectious complications, frequency of systemic antibiotic treatment was comparable in both study groups. In the treated group, three out of the 6 patients (50%) with ERC-related infectious complications received systemic antibiotic treatment. In the control group, eight out of the 14 patients (57%) with post-ERC infections were treated with systemic antibiotics at time of ERC.

The main known factors that influence the rate of post-ERC infectious complications are acute cholangitis prior to ERC and the completeness of biliary drainage [6]. At the time of ERC, 59.5% (50/84) cases of our study population displayed acute cholangitis. Incidence of acute cholangitis was similar in both study groups (*p* = 0.37). Univariate logistic regression analysis revealed a positive correlation between acute cholangitis prior to ERC and the incidence of ERC-related infectious complications (OR = 4.214; 95% CI: 1.034–17.173; *p* = 0.045). Incomplete drainage is considered as the main reason for administering prophylactic systemic antibiotic treatment in ERC. The ERC success rate of complete drainage achieved in our study was comparatively low (41.7%). Success rate was similar in both study groups (*p* = 0.07). In contrast to previous studies, we could not detect a significant benefit of successful ERC for prevention of infectious adverse events (OR = 0.368; 95% CI: 0.101–1.337; *p* = 0.13). After adjustment for the confounders “cholangitis” and “ERC success rate”, the beneficial effect of antimicrobial agents applied to contrast media for the prevention of ERC-related infectious complications was even more evident (OR = 0.153; 95% CI: 0.039–0.598; *p* = 0.007).

### Secondary sclerosing cholangitis was the most eligible biliary disorder profiting from intraductal antimicrobial prophylaxis

**Fig. 2:**
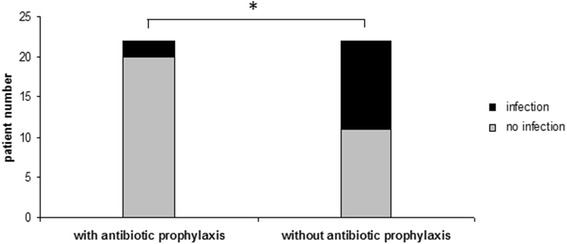
Subgroup analysis of patients with sclerosing cholangitis (SC). Addition of antimicrobial agents to ERC contrast media in patients with SC resulted in a significant decrease in infectious complications after ERC (9% with antibiotics vs. 50% without antibiotics, *p* = 0.03)

Secondary sclerosing cholangitis (SC) was the predominant etiology of biliary obstruction in our study population (52.4% of all cases). SC represents a progressive disease characterized by fibrosis and destruction of the biliary tract system leading to biliary cirrhosis. SC in critically ill patients (SC-CIP), known to be associated with a particularly rapid and aggressive progression to liver cirrhosis [24], was the most common cause of SC in our study population (32/44, 72.7%). Other causes of SC were ischemic cholangiopathy after liver transplantation (3/44, 6.8%), immunologic (4/44; 9.1%), toxic (2/44, 4.5%), infectious (1/44, 2.3%) and unknown (2/44, 4.5%). Subgroup analysis revealed that 65% (13/20) of the patients with post-ERC infection suffered from SC. When adding antimicrobial agents to ERC contrast media in patients with SC, we noted a significant decrease in infectious complications after ERC (2/22, 9% vs. 11/22, 50% in SC patients not given antibiotics into the contrast media; *p* = 0.03; Fig. 2). Furthermore, 77% (10/13) of the SC-patients with ERC-related infectious complications received a systemic antibiotic treatment before and at time of ERC. Moreover, 80% (8/10) of these SC-patients had no local antibiotic prophylaxis and developed post-ERC infectious complications despite a systemic antibiotic treatment.

## Discussion

The presented study demonstrates several important findings that may give cause to modify the current practice of antibiotic prophylaxis to prevent ERC-related infectious complications. These include: (1) addition of antimicrobial agents into the ERC contrast media significantly reduces the incidence of post-ERC infection in patients with biliary obstruction; (2) combination of different antibiotics and antifungal regiments might be even more effective; (3) the benefit of local application of antimicrobials into obstructed bile ducts is most obvious if cholangitis is already present before ERC; (4) secondary sclerosing cholangitis represents the most eligible biliary disorder which takes particular profit from locally administered antimicrobials during ERC.

The routine administration of systemic antibiotic prophylaxis to all patients undergoing ERC has been left in favor of a selective use only in those patients with suspected or known biliary obstruction for whom complete endoscopic drainage will presumably not be achieved. This concerns particularly patients with hilar strictures and PSC [19]. Patients with post-transplant biliary strictures undergoing ERC represent other feasible candidates for systemic antibiotic prophylaxis [21]. Since systemically administered antibiotics poorly penetrate into the bile in case of biliary obstruction [20, 25, 26], a theoretical benefit of injecting antimicrobial agents directly into the bile tracts during ERC is assumable. Several studies have investigated the effect of antibiotics applied in contrast media on preventing post-ERC cholangitis with conflicting results. *In vitro* studies have proven that aminoglycosides retain their antibacterial properties when mixed to ERC contrast media. Thus, the aminoglycosides tobramycin and gentamicin efficiently eliminated common biliary bacteria such as *E. coli*, *Klebsiella pneumonia*, *Proteus vulgaris* and *Pseudomonas aeruginosa* [21, 27]. In line with these findings, we observed a significantly reduced post-ERC infection rate in patients with biliary obstruction when antimicrobial agents were added into the ERC media. Patients not receiving intraductal antibiotics into ERC contrast media exhibited a 2.33-fold increased risk to develop post-ERC cholangitis.

In contrast to our results, 3 prior prospective randomized-controlled studies could not demonstrate a beneficial effect on the rate of post-ERC infectious complications by adding antibiotics into ERC contrast media [23, 28, 29]. To explain these discrepancies, one has to take into account that only an aminoglycoside was used in the three studies, and that the analyzed study population strongly differed in matters of endoscopic procedures and subtype of biliary disorders. In the two randomized controlled studies published in 1980 and 1986 [28, 29], 51% of the study population underwent solely diagnostic ERC and did not exhibit any biliary disorder, whereas in our study all patients suffered from mainly severe obstructive biliary disease. In the most recent study [23] 114 patients with non-calculous biliary obstruction were enrolled, 57 of them receiving gentamicin 10 mg into ERC contrast media. In addition, all of them received a peri-interventional systemic antibiosis. Biliary obstruction was mainly caused by malignant strictures (79% of cases vs. 9.5% in our study), whereas in our study sclerosing cholangitis (75%) was the most prevalent cause. In contrast to our study, all patients underwent endoscopic biliary stenting (vs. 9.5% in our study). Biliary obstruction was relieved resulting in an adequate drainage in all patients, whereas in our study only in 49.3% of therapeutic ERC adequate drainage was achieved. In the mentioned study, no significant difference in the incidence of post-ERC cholangitis in each group with and without gentamicin added to contrast media (8.8% each) was detected. In contrast, in our study the incidence of post-ERC infection was significantly lower when adding antimicrobial agents into the ERC contrast media (14.3% vs. 33% in the control group; *p* = 0.045). The absolute risk reduction was 19% when adding antimicrobial agents into the ERC contrast media. We suggest that patients with secondary sclerosing cholangitis, who presented 52.4% of our study population, are particularly prone to post-ERC infectious complications. Presumably, the ERC-related infectious risk in these patients is even more pronounced than in patients with malignant strictures. Thus, 65% (13/20) of the patients with post-ERC infection suffered from SC in our study. SC is a chronic cholestatic biliary disease characterized by PSC-like biliary lesions apparent on ERC, namely multifocal biliary strictures with interposed normal or dilated bile ducts [24]. The most frequent cause (72.7%) of SC in our study population was SC in critically ill patients (SC-CIP). SC-CIP is an emerging disease entity with unfavorable outcome, mostly observed in patients who have survived life-threatening illnesses and who received aggressive treatment on intensive-care units. The median survival of patients with SC-CIP who are not liver-transplanted was reported to be only 13 months [30]. ERC reveals severe bile-duct damage with extensive biliary casts and multiple irregular strictures. Recurrent episodes of bacterial cholangitis are typically observed in patients with SC [31, 32]. In 68.2% of our patients with SC, bile fluid was tested positive for bacteria before ERC with no statistical difference between the two study groups. However, infectious complication rate after ERC was significantly higher in patients with SC not given antibiotics into the contrast media (50% vs. 9%; *p* = 0.03). In these SC-patients, ERC-related infectious complications were observed even despite a systemic antibiotic treatment. Regarding the other subgroups of the study population, addition of antibiotics to the contrast media seemed to have no effect on the post-ERC infectious rate, although patient numbers are too small for valid statistical analysis.

Patients presenting with fever or elevated leucocytes prior to ERC were excluded from all previous studies that evaluated the benefit of intraductal antibiotics on post-ERC complications [23, 28, 29]. In contrast, 59.5% of our study population suffered already at the time of ERC from acute cholangitis (defined as bacterial colonization of the biliary system and elevated leucocytes > 12 000/μl/CRP > 5 mg/l). Acute cholangitis at the time of ERC was present in both, the case- and control group without statistical difference. Injection of ERC contrast media into obstructed and infected bile tracts will most likely result in bacteremia [8]. This will particularly be the case when complete biliary drainage is not achieved by ERC. On the other hand, addition of antimicrobial agents to the ERC contrast media should reduce biliary bacterial growth and decrease the risk of bacteremia. Indeed, acute cholangitis, present at the time of ERC, was calculated as a risk factor for developing post-ERC infectious complications in our study. Hence, the risk of infectious complications after ERC was 2.72-fold increased when acute cholangitis was present compared to patients without cholangitis. The absolute risk reduction was 29,3% in patients with cholangitis when adding antibiotics to contrast media. In line with our data, Motte et al. identified leukocytosis and prior cholangitis as significant risk factors for septicemia following endoscopic biliary stenting of biliary obstruction [6].

Most patients in our study received a combination of antimicrobial agents into the ERC contrast media. Only 16.6% received solely gentamicin, as used in the previous studies [23, 29]. The most frequently isolated organism in bile samples taken from patients given intraductal antibiosis were gram-positive with *Enterococcus spp.* found in 71.4%. Gram-negative organisms found in the collected bile samples were *E. coli* in 25.7%, *Klebsiella spp*. in 11.4% and *Pseudomonas spp*. in 11.4%. Of note, only in 10.7% of positive bile cultures, all detected bacterial strains were sensitive to gentamicin. Combination of gentamicin with vancomycin increased the response rate to 89.3%. These data question the effectiveness of adding solely gentamicin into ERC contrast media for prevention of post-ERC infectious complications. Instead, the choice of the administered antibiotic regiments should be based on the sensitivity of the isolated bacteria and the local pattern of antibiotic resistance. Noteworthy, we found *Candida* species in 25.7% of the fungal cultures of taken bile samples. All Candida species were sensitive to fluconazole. *Candida spp.* were shown to be predominantly detected in bile fluids of patients with primary and secondary sclerosing cholangitis, immunosuppressive therapy, after placement of plastic biliary stents, and after liver transplantation [33–36]. Our data on fungal bile cultures are in line with these findings, as our study population comprises all the mentioned entities above. In conclusion, collection of bile fluid during ERC for microbiological analysis should be considered in all patients with a high risk for post-ERC infectious complications. When adding antimicrobial agents into ERC contrast media, we recommend a combination of antibiotic and antimycotic agents instead of mono-therapy suggesting a more potent effect on preventing post-ERC infectious complications.

The main limitations of our study are the retrospective study design and the rather small number of patients. Moreover, the combination of antimicrobial agents added to the contrast media was not standardized in a uniform protocol, but was recommended to the respective investigator. This explains the number of patients receiving solely gentamicin, or an antibiotic regiment without antimycotic agents. Despite these limitations, our data are of particular interest for the clinical practice of antibiotic prophylaxis in ERC. This is the first study that evaluates ERC-related infectious complications in patients with SC. Pre-procedural cholangitis and incomplete endoscopic drainage due to multifocal biliary strictures are common findings in patients with SC, defining them as a high risk-population for post-ERC infectious complications. Injection of ERC contrast media might increase the intraductal pressure and incomplete drainage of already infected bile might then facilitate bacteremia in SC. A benefit of locally applied antibiotic agents is therefore highly assumable. Our preliminary data should now be prospectively evaluated in a larger patient cohort to improve the safety of ERC, especially in patients with SC.

## Conclusion

Based on our study results, we recommend the local application of antimicrobial agents into ERC contrast media especially in patients with SC in addition to the established systemic antibiotic prophylaxis.

## Abbrevations

CI: Confidence interval
ERC: Endoscopic retrograde cholangiography
OR: Odds ratio
PSC: Primary sclerosing cholangitis
SC: Secondary sclerosing cholangitis
SC-CIP: Secondary sclerosing cholangitis in critically ill patients

## References

[1] Andriulli A, Loperfido S, Napolitano G, Niro G, Valvano MR, Spirito F, Pilotto A, Forlano R (2007). Incidence rates of post-ERCP complications: a systematic survey of prospective studies. Am J Gastroenterol.

[2] Salminen P, Laine S, Gullichsen R (2008). Severe and fatal complications after ERCP: analysis of 2555 procedures in a single experienced center. Surg Endosc.

[3] Beilenhoff U, Neumann CS, Rey JF, Biering H, Blum R, Cimbro M, Kampf B, Rogers M, Schmidt V, ESGE Guidelines Committee, European Society of Gastrointestinal Endoscopy, European Society of Gastroenterology and Endoscopy Nurses and Associates (2008). ESGE-ESGENA Guideline: cleaning and disinfection in gastrointestinal endoscopy. Endoscopy.

[4] Dumonceau J-M, Tringali A, Blero D, Devière J, Laugiers R, Heresbach D, Costamagna G, European Society of Gastrointestinal Endoscopy (2012). Biliary stenting: indications, choice of stents and results: European Society of Gastrointestinal Endoscopy (ESGE) clinical guideline. Endoscopy.

[5] Banerjee S, Shen B, Nelson DB, Lichtenstein DR, Baron TH, Anderson MA, Dominitz JA, Gan SI, Harrison ME, Ikenberry SO, Jagannath SB, Fanelli RD, Lee K, van Guilder T, Stewart LE, ASGE Standards of Practice Committee (2008). Infection control during GI endoscopy. Gastrointest Endosc.

[6] Motte S, Deviere J, Dumonceau JM, Serruys E, Thys JP, Cremer M (1991). Risk factors for septicemia following endoscopic biliary stenting. Gastroenterology.

[7] Freeman ML (2003). Understanding risk factors and avoiding complications with endoscopic retrograde cholangiopancreatography. Curr Gastroenterol Rep.

[8] Subhani JM, Kibbler C, Dooley JS (1999). Review article: antibiotic prophylaxis for endoscopic retrograde cholangiopancreatography (ERCP). Aliment Pharmacol Ther.

[9] Yoshimoto H, Ikeda S, Tanaka M, Matsumoto S (1989). Relationship of biliary pressure to cholangiovenous reflux during endoscopic retrograde balloon catheter cholangiography. Dig Dis Sci.

[10] Lygidakis NJ, Brummelkamp WH (1985). The significance of intrabiliary pressure in acute cholangitis. Surg Gynecol Obstet.

[11] Rerknimitr R, Kladcharoen N, Mahachai V, Kullavanijaya P (2004). Result of endoscopic biliary drainage in hilar cholangiocarcinoma. J Clin Gastroenterol.

[12] Bangarulingam SY, Gossard AA, Petersen BT, Ott BJ, Lindor KD (2009). Complications of endoscopic retrograde cholangiopancreatography in primary sclerosing cholangitis. Am J Gastroenterol.

[13] Niederau C, Pohlmann U, Lübke H, Thomas L (1994). Prophylactic antibiotic treatment in therapeutic or complicated diagnostic ERCP: results of a randomized controlled clinical study. Gastrointest Endosc.

[14] Sauter G, Grabein B, Huber G, Mannes GA, Ruckdeschel G, Sauerbruch T (1990). Antibiotic prophylaxis of infectious complications with endoscopic retrograde cholangiopancreatography. A randomized controlled study. Endoscopy.

[15] Harris A, Chan AC, Torres-Viera C, Hammett R, Carr-Locke D (1999). Meta-analysis of antibiotic prophylaxis in endoscopic retrograde cholangiopancreatography (ERCP). Endoscopy.

[16] Bai Y, Gao F, Gao J, Zou D-W, Li Z-S (2009). Prophylactic antibiotics cannot prevent endoscopic retrograde cholangiopancreatography-induced cholangitis: a meta-analysis. Pancreas.

[17] Cotton PB, Connor P, Rawls E, Romagnuolo J (2008). Infection after ERCP, and antibiotic prophylaxis: a sequential quality-improvement approach over 11 years. Gastrointest Endosc.

[18] Allison MC, Sandoe JAT, Tighe R, Simpson IA, Hall RJ, Elliott TSJ, Endoscopy Committee of the British Society of Gastroenterology (2009). Antibiotic prophylaxis in gastrointestinal endoscopy. Gut.

[19] Banerjee S, Shen B, Baron TH, Nelson DB, Anderson MA, Cash BD, Dominitz JA, Gan SI, Harrison ME, Ikenberry SO, Jagannath SB, Lichtenstein D, Fanelli RD, Lee K, van Guilder T, Stewart LE, ASGE Standards of Practice Committee (2008). Antibiotic prophylaxis for GI endoscopy. Gastrointest Endosc.

[20] Blenkharn JI, Habib N, Mok D, John L, McPherson GA, Gibson RN, Blumgart LH, Benjamin IS (1985). Decreased biliary excretion of piperacillin after percutaneous relief of extrahepatic obstructive jaundice. Antimicrob Agents Chemother.

[21] Ramirez FC, Osato MS, Graham DY, Woods KL (2010). Addition of gentamicin to endoscopic retrograde cholangiopancreatography (ERCP) contrast medium towards reducing the frequency of septic complications of ERCP. J Dig Dis.

[22] Bernadino KP, Howell DA, Lawrence C, Ansari A, Lukens FJ, Sheth SG (2005). Near absence of septic complications folowwing successful therapeutic ERCP justifies selective intravenous and intracontrast use of antibiotics. Gastrointest Endosc.

[23] Norouzi A, Khatibian M, Afroogh R, Chaharmahali M, Sotoudehmanesh R (2013). The effect of adding gentamicin to contrast media for prevention of cholangitis after biliary stenting for non-calculous biliary obstruction, a randomized controlled trial. Indian J Gastroenterol Off J Indian Soc Gastroenterol.

[24] Ruemmele P, Hofstaedter F, Gelbmann CM (2009). Secondary sclerosing cholangitis. Nat Rev Gastroenterol Hepatol.

[25] Nagar H, Berger SA (1984). The excretion of antibiotics by the biliary tract. Surg Gynecol Obstet.

[26] Mortimer PR, Mackie DB, Haynes S (1969). Ampicillin levels in human bile in the presence of biliary tract disease. Br Med J.

[27] Jendrzejewski JW, McAnally T, Jones SR, Katon RM (1980). Antibiotics and ERCP: in vitro activity of aminoglycosides when added to iodinated contrast agents. Gastroenterology.

[28] Collen MJ, Hanan MR, Maher JA, Stubrin SE (1980). Modification of endoscopic retrograde cholangiopancreatography (ERCP) septic complications by the addition of an antibiotic to the contrast media. Randomized controlled investigation. Am J Gastroenterol.

[29] Pugliese V, Saccomanno S, Bonelli L, Aste H (1986). Is it useful to add gentamycin to contrast media in endoscopic retrograde cholangiopancreatography? Prospective evaluation of 330 cases. Minerva Dietol Gastroenterol.

[30] Kulaksiz H, Heuberger D, Engler S, Stiehl A (2008). Poor outcome in progressive sclerosing cholangitis after septic shock. Endoscopy.

[31] Deltenre P, Valla D-C (2006). Ischemic cholangiopathy. J Hepatol.

[32] Sherlock S (1991). Pathogenesis of sclerosing cholangitis: the role of nonimmune factors. Semin Liver Dis.

[33] Voigtländer T, Leuchs E, Vonberg R-P, Solbach P, Manns MP, Suerbaum S, Lankisch TO (2015). Microbiological analysis of bile and its impact in critically ill patients with secondary sclerosing cholangitis. J Infect.

[34] Basioukas P, Vezakis A, Zarkotou O, Fragulidis G, Themeli-Digalaki K, Rizos S, Polydorou A (2014). Isolated microorganisms in plastic biliary stents placed for benign and malignant diseases. Ann Gastroenterol Q Publ Hell Soc Gastroenterol.

[35] Kirchner GI, Scherer MN, Obed A, Ruemmele P, Wiest R, Froh M, Loss M, Schlitt H-J, Schölmerich J, Gelbmann CM (2011). Outcome of patients with ischemic-like cholangiopathy with secondary sclerosing cholangitis after liver transplantation. Scand J Gastroenterol.

[36] Gotthardt DN, Weiss KH, Rupp C, Bode K, Eckerle I, Rudolph G, Bergemann J, Kloeters-Plachky P, Chahoud F, Büchler MW, Schemmer P, Stremmel W, Sauer P (2013). Bacteriobilia and fungibilia are associated with outcome in patients with endoscopic treatment of biliary complications after liver transplantation. Endoscopy.

